# Phytate Intake, Health and Disease: “Let Thy Food Be Thy Medicine and Medicine Be Thy Food”

**DOI:** 10.3390/antiox12010146

**Published:** 2023-01-07

**Authors:** Antelm Pujol, Pilar Sanchis, Felix Grases, Luis Masmiquel

**Affiliations:** 1 Vascular and Metabolic Diseases Research Group, Endocrinology Department, Son Llàtzer University Hospital, Health Research Institute of the Balearic Islands (IdISBa), 07198 Palma de Mallorca, Spain; 2 Laboratory of Renal Lithiasis Research, Deptartment of Chemistry, University of Balearic Islands, Health Research Institute of Balearic Islands, (IdISBa), 07122 Palma de Mallorca, Spain

**Keywords:** phytate, InsP6, phytin, Mediterranean diet, DASH diet

## Abstract

Phytate (myo-inositol hexakisphosphate or InsP6) is the main phosphorus reservoir that is present in almost all wholegrains, legumes, and oilseeds. It is a major component of the Mediterranean and Dietary Approaches to Stop Hypertension (DASH) diets. Phytate is recognized as a nutraceutical and is classified by the Food and Drug Administration (FDA) as Generally Recognized As Safe (GRAS). Phytate has been shown to be effective in treating or preventing certain diseases. Phytate has been shown to inhibit calcium salt crystallization and, therefore, to reduce vascular calcifications, calcium renal calculi and soft tissue calcifications. Moreover, the adsorption of phytate to the crystal faces can inhibit hydroxyapatite dissolution and bone resorption, thereby playing a role in the treatment/prevention of bone mass loss. Phytate has a potent antioxidation and anti-inflammatory action. It is capable of inhibiting lipid peroxidation through iron chelation, reducing iron-related free radical generation. As this has the effect of mitigating neuronal damage and loss, phytate shows promise in the treatment/prevention of neurodegenerative disease. It is reported that phytate improves lipid and carbohydrate metabolism, increases adiponectin, decreases leptin and reduces protein glycation, which is linked with macrovascular and microvascular diabetes complications. In this review, we summarize the benefits of phytate intake as seen in in vitro, animal model, epidemiological and clinical trials, and we also identify questions to answer in the future.

## 1. Introduction

The influence of nutrition is crucial for the prevention and even the cure of several diseases. In fact, healthy eating patterns and regular physical activity could prevent up to 80% of instances of coronary heart disease, 90% of type 2 diabetes (T2DM) cases and 30% of all cancers [1]. The Mediterranean diet is characterized by a high intake of plant food (e.g., fruits, vegetables, breads and cereals), beans, nuts, seeds and olive oil [2]. The traditional Mediterranean diet assumes the consumption of about 1 g of phytate per day, accompanied by an appropriate amount of other minerals and bioactive components [3]. Other diets, such as the European/American diet, can provide up to 2 g of phytate per day [4]. The administration of high doses of phytate must be accompanied by an adequate content of minerals. The Mediterranean and DASH diet provide an adequate amount of legumes, whole cereals and nuts, which are rich in phytate and other minerals, in order to not negatively affect the mineral balance [3,4]. In this way, Serra-Majem et al., 2009 [5], showed that a higher adherence to a Mediterranean diet was associated with a lower inadequate intake of zinc, iodine, vitamin E, magnesium, iron, vitamin B1, vitamin A, selenium, vitamin C and folic acid in comparison with other Western diets such as the American diet.

Myo-inositol hexakisphosphate (InsP6), also known as phytic acid or phytate, is the main phosphorus reservoir that is present in almost all wholegrains, legumes and oilseeds [4]. In plant foods, it is found as a calcium–magnesium salt, called phytin. When phytate is consumed in large amounts, by itself and without being processed/cooked, it can reduce the absorption of some minerals. This has led to phytate being classified by some authors as an antinutrient. Nevertheless, this effect is only seen in laboratory conditions, and real-world data in humans do not demonstrate mineral deficiencies induced by phytate intake. Phytate is instead considered a nutraceutical: a compound that could treat or prevent disease or disorders through a variety of bioactive (e.g., antioxidant, immunomodulatory, lipid lowering) functions [1]. These properties and multiple health benefits have been repeatedly seen in the scientific literature. The FDA classify phytin as Generally Recognized As Safe (GRAS) [4].

**Figure 1:**
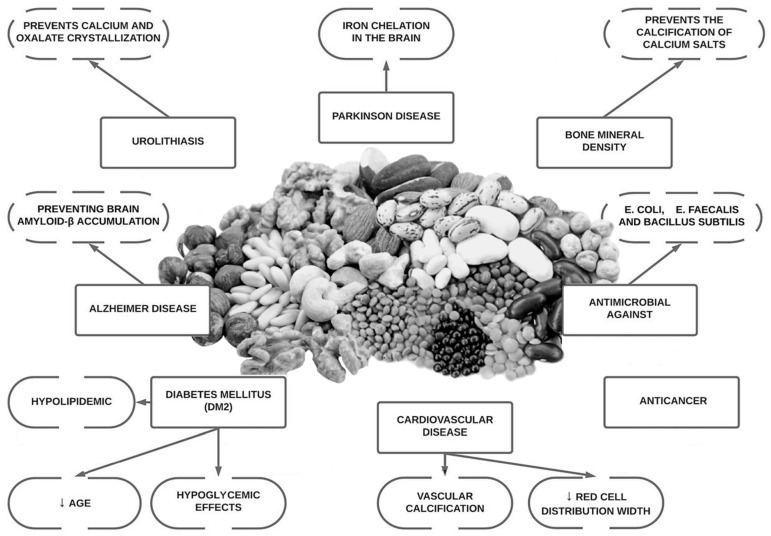
Overview of potential health benefits of phytate.

Since the discovery of the molecule phytate, research has shown that it may aid essential physiological functions, as well as offer anti-oxidant, anti-inflammatory, anti-cancer properties, be anti-diabetic, neuroprotective, antimicrobial and moreover, have the ability to prevent bone mass loss and decrease pathological calcification such as vascular calcification and renal lithiasis [4,6,7,8]. It has also demonstrated that the ingestion of phytate also generate other inositol phosphates (InsP5, InsP4, InsP3, InsP2), which can also play an important role in these pathological conditions [8]. Phytate has shown promising results in the treatment and prevention of medical conditions that have no current treatment (or where treatment options are prohibitively expensive) in in vitro data, animal models and in some human clinical trials (Figure 1). Our objective with this review is to summarize more than 50 years of research into the role of phytate in multiple health conditions and to identify questions that will need to be answered in the future.

For this narrative review, available data (from 1960 to today) were searched for in electronic databases such as PubMed, ScienceDirect and Web of Science. Searches were performed for the key words phytic acid, phytate, InsP6, IP6, inositol hexakisphosphate, phytin in combination with Mediterranean diet, DASH diet, calcifications, vascular calcifications, renal lithiasis, lithiasis, urolithiasis, osteoporosis, neurodegeneration, cognitive impairment, diabetes, type 2 diabetes mellitus, cardiovascular health, cardiovascular risk, cancer, breast cancer, anticancer properties, metabolic health and antinutrient.

## 2. Phytate and Vascular Calcification

### 2.1. Background and In Vitro Studies

Calcifications are crystalline solids made up of calcium in the human body. Some examples include renal lithiasis, dental calculus, chondrocalcinosis, calcinosis cutis and vascular calcifications [3,4,7,8]. The latter one is the result of the formation of hydroxyapatite (HAP) crystals due to excess amounts of calcium and or phosphate [5,6]. This phenomenon can be increased or decreased according to nutrient intake (vitamin D, phytate, lipids, vitamin K, etc.) or by general physical conditions or diseases (low grade inflammation, aging, chronic kidney disease or diabetes). The presence of calcifications in the arterial walls is associated with 3–4 times increased risk of coronary heart disease, stroke and heart failure [6,7,8].

Phytate has been demonstrated to inhibit calcium salt crystallization in “in vitro studies” [9]. Thus, some authors have indicated that it colud be taking place either at the nucleation level (adsorption at the surface of the nucleus) or during the subsequent growth or aggregation of the crystals, thus preventing crystallization [4,8]. Moreover, the adsorption of phytate to crystal faces can also inhibit crystal dissolution which partly explains why some agents that prevent pathological calcification, such as phytate, could also inhibit bone decalcification [9].

### 2.2. Animal Studies

Grases et al., 2008 [10], conducted a study in male Wistar rats over 66 weeks, which demonstrated that the rats who consumed phytate had significantly lower levels of calcium in the aorta compared with non-phytate rats.

Two different studies [11,12] used nicotine (generating hypertension) and vitamin D (generating hypercalcemia) to induce calcification in the renal and cardiovascular tissues of male Wistar rats. They developed significant calcium deposits in the kidneys, aorta, and heart; however, rats treated with a moisturizing skin cream containing 2.0% potassium salt of InsP6 had significantly decreased or no deposits in renal papillae, renal interstitium, renal tubules, the aorta and heart [11,12].

Similar results were obtained by Perelló et al., 2014 [13] in rats in which aggressive cardiovascular calcifications were induced by the administration of high doses of vitamin D. The rats were injected with SNF472 (an intravenous formulation of InsP6) and reductions of aortic and heart calcification by 60 and 70% were seen, respectively. This model was also used to induce calcinosis by Grases et al., 2007 [14] and Ketteler et al., 2013 [15]. The 2007 study used three high doses of vitamin D on Sprague Dawley rats showed that phytate produces a greater reduction in aortic calcium calcifications when compared with rats that had been treated with the placebo or etiodronate [14]. The 2013 study, using a similar study design, compared SNF472 with oral cinacalcet and sodium thiosulfate, proving that SNF472 reduced calcifications by 60% in the aorta and by 68% in myocardial tissue in comparation with a 24% reduction induced by cinacalcet and no reduction at all with sodium thiosulfate [14].

Another model to induce plaque formation is by using subcutaneous injection of 0.1% potassium permanganate (KMnO_4_) solution [16]. They found that rats treated with a topical application of 2% InsP6 skin cream experienced a significant reduction in plaque size in comparison with the placebo [16]. The urinary levels of phytate in the cream-treated group were significantly higher, showing that topical administration can be an effective route for phytate administration [16].

Thus, the administration of phytate, whether through the diet, topical skin application or intravenously, has been shown in animal models to be an effective treatment in the reduction in vascular calcification [11,12,13,14,15,16].

### 2.3. Epidemiological Studies in Humans

The CAC (coronary artery calcium) score derived from computerized tomography scanning is a validated tool used to assess vascular calcification. The relationship between CAC, cardiovascular risk and mortality has been widely studied in the general population, elderly people, people living with diabetes and patients living with chronic kidney disease with or without dialysis [17,18,19,20,21]. However, the high cost of the technique makes its application to a large amount of the scientific studies difficult.

Fernández-Palomeque et al., 2015 [22], in a cross-sectional study in a population of 188 elderly subjects, found that subjects with higher levels of urinary phytate had less mitral calcification. Phytate consumption was independently associated with valve calcification.

Sanchis et al., 2016 [7], in a prospective cross-sectional study carried out among sixty-nine patients with chronic kidney disease, estimated phytate consumption based on a food frequency questionnaire and quantified abdominal aortic calcification (AAC) using lumbar X-rays. Patients with no/mild AAC were younger, had lower blood pressure, lower prevalence of prior cardiovascular disease, greater dietary phytate intake and greater urinary phytate compared with patients with moderate/severe AAC. Phytate consumption was negatively associated with AAC.

Nuts and legumes are a good source of phytic acid (about 1–5% of their weight are phytin) [23]. Lichtensin et al., 2021 [24], in the latest “Dietary Guidance to Improve Cardiovascular Health”, recommended high intakes of legumes and nuts for the prevention of cardiovascular issues. Several studies identified nut intake as a protective factor against cardiovascular disease (CVD), especially for coronary heart disease as well as stroke incidents and stroke mortality [25,26].

### 2.4. Clinical Trials in Humans

Perelló et al., 2018 [27], enrolled both healthy and hemodialysis patients in the first-in-human, double-blind, randomized, placebo-controlled Phase I study to assess the safety, tolerability and pharmacokinetics of SNF472. Inhibition of the induction of hydroxyapatite crystallization in plasma samples was demonstrable by SNF472. After this first trial, more evidence emerged relating to the role of SNF472 in treating vascular calcification and calciphylaxis in hemodialysis patients [28,29]. SNF472 has completed early-phase clinical trials with a favourable safety profile, and Phase 2 clinical trial data have shown attenuation of coronary artery and aortic valve calcification in patients receiving hemodialysis [30].

Sanchis et al., 2018 [31] demonstrated in an “in vitro” and “in vivo” randomized crossover trial that consumption of InsP6 inhibits protein glycation in patients with T2DM. Advanced glycation end products (AGEs) and the receptor for advanced glycation end products (RAGE) play pivotal roles in vascular calcification in atherosclerosis [32]. AGEs contribute to microvascular and macrovascular complications in T2DM, in chronic kidney disease, and in aging-related complications [31,32]. The reduction in the production of AGEs could be an effective strategy to reduce vascular calcification.

Estruch et al., 2018 [33], in a multi-centre trial in Spain, enrolled 7447 participants at high cardiovascular risk who were assigned one of three diets: a Mediterranean diet supplemented with extra-virgin olive oil, a Mediterranean diet supplemented with mixed nuts, or a control diet. After a median follow up of 4.8 years, the incidence of major cardiovascular events was lower among those assigned to a Mediterranean diet supplemented with extra-virgin olive oil or nuts than among those in the control diet group. We could hypothesize that part of these benefits could be due to the high phytate content in nuts, and in the Mediterranean diet generally.

## 3. Phytate and Urolithiasis

### 3.1. Background and In Vitro Studies

Urolithiasis, otherwise known as kidney stones or renal calculi, is a multifactorial and highly prevalent urological condition. Its prevalence has increased in recent years and stands at 12% worldwide. In some regions in Europe, its prevalence is as high as 15% [34,35]. Most kidney stones are composed of calcium in the forms of calcium oxalate or calcium phosphate, or a mixture of both, representing 70–80% of the total. Calcium oxalate kidney stones are the most prevalent [34,35]. The process of kidney stone formation is not fully understood. It is thought that crystallization occurs when the concentration of ions exceeds their saturation point in the solution. This would occur when a solution contained a greater amount of dissolved material than could be dissolved by the solvent under normal circumstances [35].

As previously mentioned, phytate could inhibit calcium salt crystallization at the nucleation level (adsorption at the surface of the nucleus) or during the growth or aggregation of the crystals, thus preventing crystallisation [9]. Early in vitro work by Grases et al., 1988 [36], showed that the antioxidant action of phytate may play a role in reducing calcium oxalate crystallization. These results were replicated in posterior studies [12,37]. This effect could be enhanced by the addition of magnesium [38].

### 3.2. Animal Studies

Grases et al., 2004 [39], compared the effectiveness of three different phytate salts in reducing urinary calcium in rats. The most significant results were seen with the use of potassium salt. Phytate intake did not affect levels of urinary oxalate.

Grases et al., 2007 [14], induced vascular calcification in 18 male Wistar rats by engendering hypertension and hypercalcemia (using nicotine and vitamin D, respectively) while feeding them with AIN 76-A diet (phytate-free diet). One group was treated with topical skin application of 4 g of a cream containing 2.0% potassium phytate salt, resulting in significant reduction or absence of calcium deposits in kidneys and papillae, as well as in kidney tubules and vessels, compared with non-treated rats. This study showed that phytate acts as a crystallization inhibitor in the intrapapillary tissue and urine. Again, the phytate effect could be enhanced by the addition of magnesium.

Different results were reported by Kim et al., 2020 [40] in his most recent study. Four-week-old male and female Sprague Dawley rats were fed AIN-93G (phytate-free diet) supplemented with 0%, 1%, 3%, or 5% phytate for 12 weeks, with a constant supplementation of calcium (Ca2+) concentration. The trial showed that in the AIN-93G diet plus phytate supplementation caused a time and concentration dependent impairment of the renal reabsorption of Ca2+ and phosphate accompanied by parathyroid (PTH) increase, which predisposed to nephrocalcinosis development. In this case, it is very important to consider that the phytate supplement was administered in the form of sodium salt and not calcium-magnesium, which is how it is found in food.

### 3.3. Epidemiological Studies

The relationship between different dietary factors (liquid intake, pH, calcium, phosphate, oxalate, citrate, phytate, urate and vitamins) and each type of kidney stones has been extensively studied [41]. A general list of recommendations to follow in order to avoid the formation of renal calculi are: daily intake of 2 L water, avoid strict vegetarian diets, avoid excessive animal protein diets, avoid excessive salt (NaCl) consumption, avoid excessive vitamin C and/or vitamin D consumption, avoid exposure to cytotoxic substances and consume phytate-rich products (natural dietary bran, legumes and beans, whole cereals). A considerable number of epidemiological studies have identified a diet rich in phytate as a protective agent in the development of renal calculi [42,43].

The Nurses’ Health Study II [44] is a prospective study that followed 96,245 female participants over the course of eight years, all of whom were young and without any renal calculi history. Dietary habits were assessed using a food frequency questionnaire. Women in the lowest quintile of phytate intake in comparation with the highest quintile had fewer kidney stones. Supplemental calcium intake and a higher intake of animal proteins and sucrose were associated with a higher risk of calcium kidney stones.

In a cohort study [45] conducted over the course of one year 165 healthy children aged between five and 12 years old were asked to fill out a dietary questionnaire to determine their dietary habits. Phytate and citrate concentration and excretion were studied in two urine samples (baseline and a 12 h overnight sample) for each child. Low urinary concentrations of phytate and citrate were found in almost one-third of the studied population. These findings suggest that despite children being at low risk of developing kidney stones, diet is a risk factor in their possible development.

The multiple beneficial effects of the Mediterranean diet include a reduced risk of renal calcium salt and uric acid kidney stones. Prieto et al., 2019 [34], in a cross-sectional study in overweight individuals with metabolic syndrome found that good adherence to a Mediterranean diet with a high consumption of fruit, vegetables, legumes and nuts decreased the risk of kidney stones. Moreover, the Dietary Approach to Stop Hypertension (DASH) diet and the Mediterranean style diet have some similarities, particularly in food selection. The DASH diet has also been associated with reduced risk of kidney stones [46,47]. Both diets are high in phytate.

### 3.4. Clinical Trials in Humans

Conte et al., 1999 [48], selected three groups of patients with calcium oxalate kidney stones and determined their lithogenic risk through urine tests. Patients with calcium oxalate stones treated with potassium citrate or phytate diet reported a decreased rick on the ithogeny test performed by the authors at the beginning and at the end of the trial. The risk reduction was 52% and 50%, respectively.

Recently, Guimerà et al., 2022 [49], performed a controlled randomized trial on the effects in humans of daily administration of 380 mg capsule of calcium-magnesium InsP6 on calciuria. The studied population comprised adult patients with urinary stones, hypercalciuria (>250 mg/24 h) and osteopenia or osteoporosis (determined by densitometry) in the femur and/or spine. At 3 months, the phytate group had significantly lower calciuria levels.

Besides renal lithiasis, interesting results have been seen in a study administering phytate in a mouthwash solution to 25 healthy dental plaque-forming volunteers [50]. A randomized, double-blind, three-period crossover clinical study showed a statistically significant decrease in calcified dental plaque residues in the phytate-treatment period compared with control and placebo periods. The efficacy of the treatment in the prevention of calculus formation suggests another application of phytate as a crystallization inhibitor.

Clearly, there is an urgent need of more human clinical trials assessing the effect of phytate in preventing and treating kidney stones. In vitro and epidemiological studies showed the efficacy of phytate as a renal calculi inhibitor. It should be borne in mind that there is little treatment available to prevent renal calcification, a prevalent condition.

## 4. Phytate and Osteoporosis

### 4.1. Background and In Vitro Studies

Osteoporosis is a highly prevalent bone disorder especially in post-menopausal women [51]. Lifestyle interventions such as physical activity and nutrition play an important role in the prevention and treatment of bone mineral loss [9,51,52,53].

Phytate is an inhibitor of crystallization due to the capacity of binding to the crystal surface. The adsorption of phytate to the crystal faces can inhibit pathological calcifications in vivo and in vitro as mentioned, but can also inhibit hydroxyapatite dissolution and bone resorption [9]. It is important to remark that pyrophosphate and bisphosphonates were discovered previously as inhibitors of both crystal formation and dissolution by binding strongly to HAP crystals. In fact, the bisphosphonates are widely use as drugs for bone mass loss disease [54].

Phytate has shown the ability in vitro to reduce the osteoclastogenesis of human primary osteoclasts (human peripheral blood mononuclear cell culture (PBMNC) and mouse macrophage RAW264.7 cell lines) [55]. Similar results have been obtained in previous in vitro studies [56,57,58,59]. In a recent in vitro study [9], phytate was able to inhibit acid-driven HAP dissolution. Moreover, the inhibitory effect of phytate on HAP dissolution was greater than that exhibited by etidronate and similar to that of alendronate [9]. Additionally, phytate inhibited HAP dissolution in a concentration-dependent manner [9]. Phytate could help in two opposite processes: to prevent calcification and to decrease bone mass loss [9].

### 4.2. Animal Studies

In an animal model study on postmenopausal osteoporosis carried out over 12 weeks on ovariectomized rats, one group was fed with AIN-76A (no phytate diet) and the other group with AIN-76 enriched with 1% phytin. The phytin-consuming group showed an increase in bone mineral density in femoral bones and L4 vertebrae. Phytin reduced the bone density loss caused by estrogen deficiency [60].

On the other hand, Kim et al., 2020 [40], challenged the previous results. In a study using four-week-old male and female Sprague Dawley rats the systemic effects of dietary phytate were evaluated. Rats were fed AIN-93G diets supplemented with 0%, 1%, 3%, or 5% phytate over 12 weeks with a constant supplementation of calcium concentration. The trial showed that in the AIN-93G diet plus phytate supplementation caused a time and concentration dependent impairment of the renal reabsorption of calcium and phosphate accompanied by PTH increase, predisposing the rats to the development of hypophosphatemic rickets. Once again, it is necessary to consider that these authors used sodium phytate in their study, and not the calcium–magnesium salt, which is the form in which it is found in food.

### 4.3. Epidemiological Studies

The high number of epidemiological studies linking phytate consumption with an improvement of bone health is worth of mention. A descriptive cross-sectional pilot study [61] carried out among 143 postmenopausal women examined the relationship between urinary phytate concentration and risk of fracture within 10 years (using the FRAX model). The risk of major osteoporotic fracture and hip fracture were higher in women with low urinary phytate levels. This difference was higher in women with at least one risk factor for osteoporosis. Similar results were obtained previously by Lopez-González et al., 2013 [62], in a study carried out among 157 postmenopausal women; low InsP6 levels were associated with significantly greater bone mass loss in the lumbar spine and the 10-year fracture probability (calculated by the FRAX model) was also significantly higher in the low-phytate group compared with the high-phytate group, both in hip and major osteoporotic fracture [62]. Moreover, in a prospective study with 1473 subjects they found that the higher the phytate consumption, the greater mineral density [63]. Similar results with a similar design study were found by Lopez-González et al., 2011 [64], where phytate consumption was measured by food questionnaires and bone mineral density was evaluated by dual-X-ray double-energy absorptiometry. The results indicated that adequate phytate consumption may play a significant role in the prevention of bone mineral density loss in postmenopausal women [64].

Sanchis et al., 2021 [9], carried out a cross-sectional study in which 415 women completed a validated 14-item questionnaire designed to estimate adherence to the Mediterranean Diet and phytate consumption and where bone mineral density of the L1-L4 was evaluated by dual energy X-ray absorptiometry (DXA). The results showed a significant association between low phytate consumption and low bone mineral density at lumbar spine [9]. According to these data, the ingestion of at least 307 mg/day would prevent bone mineral loss. In a practical sense, this would be highly achievable since the Mediterranean diet is associated with a phytate intake of 1–2 g per day [31,65].

In this sense, the Mediterranean diet has been demonstrated to be an effective method to increase bone mineral density. As mentioned before, diet is a modifiable factor which is crucial to reduce the risk of osteoporosis [66]. The Mediterranean diet, along with other diets such as DASH, is rich in fruits, vegetables, legumes and nuts, all of which are associated with better bone health in epidemiological studies [52,66,67,68].

### 4.4. Clinical Trials in Humans

As mentioned previously, Guimerà et al., 2022 [49] conducted the first controlled randomized trial on the effects in humans of daily phyate supplementation on bone mineral density (BMD) as a secondary outcome. They used ß-Crosslaps as a serum maker for predicting BMD and the response to antiresorptive treatment. Patients with hypercalciuria (>250 mg/24 h) and osteopenia or osteoporosis (determined by densitometry) in the femur and/or spine who received daily administration of 380 mg capsule of calcium–magnesium InsP6 had significantly lower levels of ß-Crosslaps in comparation with the placebo group after three months of supplementation.

## 5. Phytate Cognitive Function and Neurodegenerative Disease

### 5.1. Background and In Vitro Studies

Maintaining or achieving normal and optimal cerebral function is a subject of interest for people wishing to increase their cognitive performance (students, workers, athletes, etc.) and wishing to reduce their risk of development of neurodegenerative disease. Cognitive performance and cognitive decline are multifactorial [69]. Physiological processes such as calcium homeostasis, mitochondrial dysfunction, oxidative damage, systemic inflammation, and increased susceptibility to stresses might be hallmarks of cognitive decline [68].

Nutritional choices may play a role in brain health. Food groups (fruits, vegetables, cereals and grains) and nutrients (zinc, selenium, copper, fiber, some vitamins, phytochemicals and polyphenols) have been identified as protective agents against cognitive decline and also cognitive performance enhancers [69,70,71].

Brain tissue is very susceptible to oxidative stress because of the high levels of polyunsaturated fatty acids, low antioxidant concentrations (superoxide dismutase and catalase are lower than in liver tissue) and the high oxidative stress environment [72]. Phytate and the products that result from its metabolism (from InsP5 to InsP2) can exhibit potent antioxidation and anti-inflammatory action. It blocks catalyzed hydroxyl radical (OH-) formation [72,73], inhibits lipid peroxidation [72,74], and minimize iron-related free radical generation, hence mitigating neuronal damage and loss [72,73,74]. Moreover, InsP6 but not InsP3, InsP4 or InsP5 inhibits the activity of amyloid-β precursor protein (BACE1), a protein involved in amyloid-beta accumulation. BACE1 inhibition could prevent Aβ accumulation [75]. All these results have been obtained in in vitro models.

### 5.2. Animal Studies

Phytate can be delivered to the brain. Grases et al., 2007 [76] have demonstrated in a rat model study that phytic acid can cross the blood–brain barrier efficiently, as a ten-fold increase in phytic acid concentrations have been seen in rat brains compared to other tissues after a high phytic acid diet.

Alzheimer’s disease is a highly prevalent progressive neurodegenerative disorder. Accumulation of beta-amyloid in the brain has been implicated in the physiopathology. Anekonda et al., 2011 [77] evaluated the protective effects of phytate against amyloid beta in Tg2576 mouse model where intraneuronal beta-amyloid accumulation was increased. Over the course of 6 months, female rats were treated with 2% phytic acid drinking water or placebo. No effects from copper, iron and zinc were found on brain levels. Nevertheless, phytate had modest anti-amyloid effects, as well as effects on potentially novel therapeutic targets (SIRT1, PAMPK, autophagy and vesicle proteins). The dose of 2% can be considered rather mild, as legumes, cereals, oil seeds and nuts provide 1–5% phytic acid in the Mediterranean diet. Phytate was also well tolerated. The authors hypothesized that phytate intake mimics caloric restriction, promoting autophagy (activation of the AMPK pathway) and modulating clathrin-coated endocytosis of amyloid-β precursor protein (APP) and its cleavage products.

Parkinson’s disease is the second most prevalent neurodegenerative disorder after Alzheimer’s disease. Excess iron accumulation has been established as one of the most important histopathological and pathophysiological processes related to Parkinson´s disease. Phytate intake was able to reduce 6-hydroxydopamine (6-OHDA) induced apoptosis in both normal and excess iron conditions in cell culture models [73]. These results were replicated in (6-OHDA)-induced Parkinson´s disease in Wistar rats [74]. Significantly reduced rotations and motor asymmetry in 6-OHDA lesioned rats were seen in the rats that received an oral phytate pre-treatment [74].

### 5.3. Epidemiological Studies

Diets known to be high in phytate are associated with lower cognitive decline. In a cohort study [78] of 106 patients, who had undergone annual cognitive tests and who had clinical histories of strokes, a food frequency questionnaire was used to find out their dietary habits. Those who followed a diet rich in wholegrains, leafy greens and other vegetables, beans and nuts were found to have had a slower rate of global cognitive decline over an average of 5.9 years of follow-up [78]. This combination of food types is typical of Mediterranean and DASH diets which are also rich in phytates [33,34]. In fact, Van der Brick et al., 2019 [79], in an extensive review, support higher adherence to the Mediterranean diet, DASH diet or a combination of the two as a measure to prevent cognitive decline and Alzheimer´s disease [79].

The association between phytate intake and prevention of cognitive decline and neurodegenerative disease has been found. Recently, Larvie et al., 2021 [69], used data from the 2013–2014 National Health and Nutrition Examination Survey (NHANES) and the corresponding Food Patterns Equivalents Database (FPED) to study the association of phytate intake and cognitive function. They found that in adults over 60 years old, after controlling other covariants, daily phytate intake was positively associated with cognitive function.

These results were not exclusive to elderly populations. Cormick et al., 2019 [80] followed 835 children between the ages of 6 months and 60 months (five years) in order to find factors that were associated with higher scoring trajectories. Phytate intake had a significant association with higher academic and cognitive performance.

### 5.4. Clinical Trials in Humans

So far, there is no randomized clinical trial assessing the effect of phytate intake in cognitive decline and neurodegenerative disease.

## 6. Phytate Intake, Type 2 Diabetes Mellitus and Cardiovascular Health

### 6.1. Background and In Vitro Studies

T2DM is an endocrine disorder that is characterized by hyperglycemia with alterations in carbohydrate, protein and fat metabolism. Microvascular and macrovascular complications are the main concerns in patients living with poor T2DM control. The 2022 American Diabetes Association Standards of Care focuses on treating T2DM with a metabolic-centric approach and not just a glucose-centric approach [81]. Most of the people living with DM2 are patients with high or very high cardiovascular risk [82]. Controlling hyperglycemia and managing other cardiovascular risk factors would be the goal of the patient centered treatment [81].

Sustained and uncontrolled hyperglycemia produces changes in cell membrane permeability and transmembrane potential, influencing the relationship of the cell with the environment [83]. Thus, hyperpolarization induces glucose oxidation [84], protein glycation [85,86], activation of the polyol pathway, and increased oxidative stress, leading to a state of low-grade inflammation and pro-oxidative state [83,84,85,86,87].

Phytate intake can play a role in controlling hyperglycemia but, more importantly, also in reducing cardiovascular risk by different mechanisms. Alterations in inositol metabolism (inosituria and inositol intracellular depletion) have been associated in several human and animal studies with hyperglycaemia and insulin resistance [88]. Phytate would reduce oxidative stress by acting as an iron chelator, preventing the generation of iron-driven hydroxyl radical formation and decreasing lipid peroxidation [72,73,74]. InsP6 and the formation of lowers forms (via degradation of InsP6) especially InsP3 play an important role in insulin secretion by regulating calcium-homeostasis [89,90]. Amylase inhibition activity by phytate has been described and would reduce the rate of carbohydrate digestion and absorption [91]. Moreover, phytate could exercise its positive effects too by decreasing leptin and increasing adiponectin levels [89]. On one hand, leptin action promotes an increase in the drive for food, reduced satiety, and energy utilization [88]. On the other hand, higher adiponectin concentrations produce an antioxidant response and are associated with a decreased level of C-reactive protein and interleukin-6 (IL-6) [88].

InsP6 influences lipid metabolism. Researchers have reported a reduction in lipase activity, total cholesterol, low-density lipoprotein, hepatic total lipids, and hepatic triglycerides, whereas increasing high-density lipoprotein levels are also seen in InsP6 supplementation [88]. The salt type of phytate administration will be crucial to the outcome lipid levels. The sodium-phytate form decreases cholesterol concentrations, whereas the calcium–magnesium form can increase cholesterol concentrations [88]. It is hypothesized that the calcium–magnesium form would not bind to bile acids, reducing fecal bile excretion [92].

Protein glycation leading to the accumulation of AGEs is thought to be one of the main factors triggering the diabetic complications, including nephropathy, retinopathy and neuropathy [92,93,94,95,96,97]. AGE accumulation alters the intracellular signalling and gene expression and releases pro-inflammatory molecules and free radicals [93,94,95,96,97,98]. In fact, proteins are not the only molecules that can produce AGEs as other endogenous components, lipids or nucleic acids can also lead to AGE formation [93,94,95,96,97,98]. Sanchis et al., 2018 [31], showed that InsP6 significantly reduces AGE formation, and in a dose-dependent manner, because InsP6 can strongly chelate Fe3+, preventing the subsequent formation of free radicals [31].

Red cell distribution width (RDW) is a numerical measure of the amount of variability in red blood cell size, which is routinely used in the differential diagnosis of anaemia and has been suggested as a predictor of cardiovascular diseases and anaemia [88]. Inflammation might increase RDW levels through the impairment of iron metabolism [99]. InsP6 could reduce RDW through anti-inflammatory and antioxidant effects [88].

**Figure 2:**
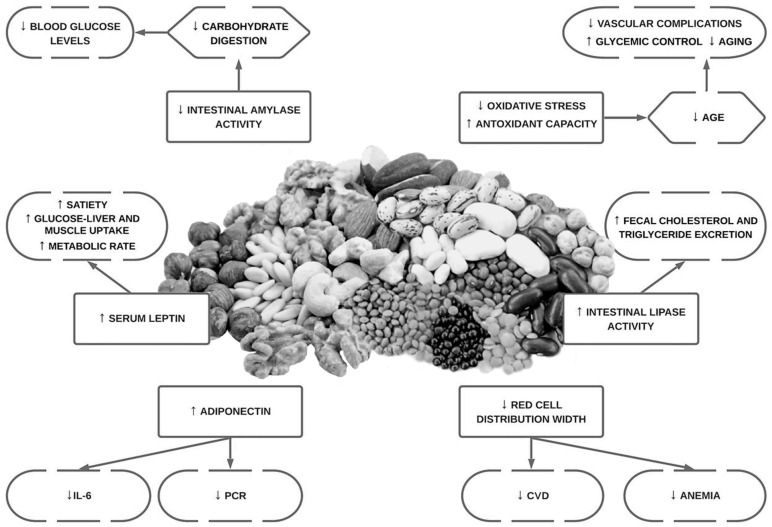
Overview of the potential bioactivities in cardiovascular health of phytate.

Hyperuricemia it is considered a risk factor for cardiovascular events, and it is the most frequent cause of acute arthritis in men [100]. Even countries where the local population had historically low levels of serum uric acid, have experienced an increase in serum uric acid concentration (SUA) due to the acquisition of a Western diet eating pattern [100]. Restricting dietary purine intake should be an effective method for maintaining fasting SUA. However, restricting purine intake in the long term could be difficult [100]. InsP6 has been shown to inhibit purine metabolism in vitro by competitively inhibiting the hydrolysis of purine nucleotides [100]. An overview of the potential cardiovascular bioactivities of phytate can be seen below (Figure 2).

### 6.2. Animal Studies

Dilworth et al., 2005 [89], in a comparative study in rats that were fed phytate plus zinc, phytate alone, zinc alone or placebo, the activities of enzymes involved in carbohydrate and lipid metabolism, as well as transaminases in the liver were assessed. The phytate came from two different sources: phytic acid extracted from sweet potato (Ipomea batatas) or commercial phytic acid. Phytic acid lowered blood glucose (seen with both phytate sources) and increased the activity of glucose-6-phosphate dehydrogenase (this was only seen with phytic acid extracted from sweet potatoes). NADPH generation by glucose-6-phosphate dehydrogenase was used by glutathione reductase to maintain reduced glutathione levels. The promotion of an antioxidant environment could be one of the explanations as to why the up-regulation of the glucose-6-phosphate dehydrogenase activity by phytate could reduce insulin resistance [87]. Similar results were previously found by Onomi et al., 2004 [101], in rats fed a high-sucrose diet. A high-sucrose diet and an amount of phytate ranging from 0.2% to 10% were provided [101]. Rats who ingested 10% sodium phytate experienced a reduction in lipogenic enzymes and lower growth, food intake, serum triglyceride and cholesterol levels, in a dose dependent manner [101]. Lee et al., 2006 [102], reported that diabetic KK mice who were fed purified diets supplemented with different concentrations of sodium phytate (0, 0.5 and 1%) over eight weeks reduced calorie intake, body weight, levels of fasting and random blood glucose, glycated hemoglobin (HbA1c) as well as insulin levels [102].

Phytate can cause inhibition, in a dose-dependent manner, of α-glucosidase and α-amylase activity comparable to standard drug acarbose, in vitro and in rats with streptozotocin–nicotinamide-induced type 2 diabetes mellitus [103]. The antidiabetic function of phytic acid may work in part through the decrease in the activity of intestinal amylase which is indicative of lesser products of carbohydrate digestion formation and subsequently absorption, leading to a decreased percentage spike in random blood glucose [103,104].

A high-fat diet and streptozotocin is a model used to induce T2DM in Sprague Dawley rats. Omoury et al., 2013 [104] used this model to study the effects of the combination of InsP6 and inositol, in comparation with glibenclamide, on several markers of metabolic health over four weeks. The combination of InsP6 and inositol in the mentioned dose achieved better glycemic control (reduced blood glucose plus reduce HOMA-IR index) than glibenclamide. Interestingly, this was the first study to show that leptin levels were increased by phytate [104]. The increased concentration of leptin can explain why, in this study, the rats treated with phytates reduced their food intake by 45%. The effects of phytate intake on lipid metabolism were as expected: reduction in triglycerides and total cholesterol as seen in various works [101,104]. A later study [105], using a very similar approach, found that the increase in serum *α*-amylase activity in diabetic rats treated with combined InsP6 and inositol or glibenclamide was not significant compared with (that of) the nondiabetic control group. Low serum *α*-amylase concentrations are associated with pancreatic exocrine-endocrine disorders. The authors hypothesized that the nonsignificant increase in serum *α*-amylase activity in diabetic rats treated with combined InsP6 and inositol or glibenclamide, compared with the nondiabetic control group, might restore the metabolic abnormalities caused by T2DM. A decreasing trend in the Na^+^/K^+^ ATPase activity in the group treated with combined InsP6 and inositol supplement could reduce intestinal carbohydrate absorption. Moreover, in this later study, the authors found that a reduction in RDW levels in the diabetic rats treated with the InsP6 and inositol or glibenclamide can reduce cardiovascular risk.

### 6.3. Epidemiological Studies in Humans

Mediterranean and DASH diets have repeatedly shown their effectiveness in improving glycemic control and decreasing cardiovascular risk. Furthermore, these diets are rich in phytates [33,34]. In the latest “Dietary Guidance to Improve Cardiovascular Health” the Mediterranean diet and the DASH diet are recommended to reduce cardiovascular disease risk (CVD) [24]. The ATTICA study [106] revealed that those who had a higher adherence to a Mediterranean diet improved fasting glucose homeostasis, insulin levels and a lower insulin resistance index (HOMA) in both normoglycemic and diabetic patients [106]. Several works proved that adherence to the Mediterranean diet exerts a protective effect against loss of glycaemic control [106]. These effects on cardiovascular health and glycemic control could be produced by the anti-oxidation and anti-inflammation effects of the Mediterranean diet [107].

In a more specific way, certain foods present in the Mediterranean diet have been investigated in isolation. Several studies proved that a higher intake of legumes and nuts for cardiovascular prevention [24,25,26]. Nuts are identified as a protective factor against cardiovascular disease, especially coronary heart disease and stroke incidence and mortality [22,26]. Improvements in glycaemic control and a reduction in HbA1c were reported as a result of increased dietary intake of legumes and wholegrains [107,108,109]. High intake of dietary fibre, specifically of the soluble type, improves glycaemic control, decreases hyperinsulinemia, and lowers plasma lipid concentrations in patients with type 2 diabetes [108].

Legumes and whole grains are rich in InsP6 and in fiber; this could explain, to a certain extent, the benefits reported in the scientific literature. Sanchis et al., 2018 [31], mentioned that observational evidence suggests that in the Mediterranean region, the InsP6 consumption is lower in patients with T2DM than in non-diabetic subjects (unpublished data) [31].

### 6.4. Clinical Trials in Humans

Most clinicals trials published in this regard investigate the effects of the Mediterranean diet or DASH diet on T2DM or cardiovascular health. The multi-centre, randomized, primary prevention trial of cardiovascular disease (Prevención con Dieta Mediterránea “PREDIMED” Study) [110] in 772 asymptomatic patients, aged between 55 and 80 years of age at high cardiovascular risk, saw improved fasting blood glucose, reduced blood pressure and increased high-density lipoprotein (HDL)/cholesterol ratios in these diets in comparison with a low-fat diet. The participants did not lose weight on either diet [110]. Toobert et al., 2013 [111] tested the effectiveness of the Mediterranean Lifestyle Program (MLP) in 279 post-menopausal women. After six months of intervention a reduction in 0.4% units in HbA1c was reported [111]. In a one-year randomized trial of 259 patients people living with T2DM, a low-carbohydrate Mediterranean diet, a traditional Mediterranean diet, and an American Diabetes Association (ADA)-proposed diet were compared. The low-carb Mediterranean diet and the traditional Mediterranean diet showed better weight loss effects and better reduction in HbA1c compared with the other diet [112].

The effectiveness of certain high-phytate foods in improving cardiovascular health has been tested in clinical trials. Recently, 31 people living with T2DM were randomly assigned to two different groups: one designated to consume a legume-free diet, the other to consume a legume-based diet for 8 weeks. Legumes significantly increased serum adiponectin concentrations [113].

Because it is difficult to elucidate or discriminate the effects of phytate from the other components of the diet, clinicals trials using only InsP6 are much needed. In fact, Sanchis et al., 2022 [114] in a randomized crossover trial they provided to people living with T2DM 1 capsule of 380 mg of calcium-magnesium InsP6 twice daily during 12 weeks. When patients received InsP6 supplementation they had significant decrease serum levels of HbA1c and increase adiponectin levels. However, no differences were found in IL-1beta, IL-6 and tumor necrosis tumor necrosis alpha (TNF-alpha). This work proves for the first time that phytate supplementation could increase adiponectin levels in patients living with T2DM.

Sanchis et al., 2018 [31] for the first time reported the inhibitory effect of InsP6 on protein glycation, reducing both in vitro and in vivo AGEs in patients living with T2DM. In this randomized cross-over trial [31], 35 patients received either an InsP6 diet (diet plan plus one capsule of 380mg of calcium-magnesium InsP6) or a non-InsP6 diet (the same diet plan without InsP6 supplementation). When the subjects took the InsP6 supplementation, they experienced a reduction of 25% of the levels of circulating AGEs and a 3.8% decline in HbA1c, probably because of reduced overall protein glycation.

Ikenaga et al., 2019 [100] in a randomized double-blind placebo-controlled trial assessed the effect of the repeated intake of InsP6 on fasting SUA levels in hyperuricemic subjects, and demonstrated that two weeks of supplementation with twice-daily 600 mg of InsP6 improved fasting SUA levels in these subjects [100].

## 7. Phytate Intake and Cancer

### 7.1. Background and In Vitro Studies

Living with overweight or obesity is associated with increased risk (a 11.9% in men and 13.1% in women) for a range of malignancies in at least 13 anatomical sites [115]. Insulin resistance and a poor metabolic profile could lead to low-grade inflammation and oxidative stress, two physiopathological drivers of the association between excess body weight and cancer [115]. Nutrition plays an important role in the prevention and even the treatment of cancer.

Phytate anti-cancer activity is not fully understood [116]. Phytate can reach cancer cells [116]. It is hypothesized that the involvement of lower phosphate inositol phosphates (InsP1-3) in signal transduction pathways can affect the cell cycle regulation, growth, and differentiation of malignant cells [117]. The iron chelation effect and the suppression of hydroxyl formation, explains the antioxidant effect of phytate intake and this could have a role in reducing the low-grade inflammation which is a hallmark of cancer [69,73,117,118].

Since 1998, the scientific evidence shows that phytate could inhibit pathways involved in malignancy such as cell proliferation and growth, metastasis, angiogenesis, apoptosis and differentiation [118,119,120,121,122]. The effects produced by phytate could benefit patients under chemotherapy treatment by reducing side effects correlated to the treatment, thus improving the patient’s quality of life and even improve long-term survival [117].

**Figure 3:**
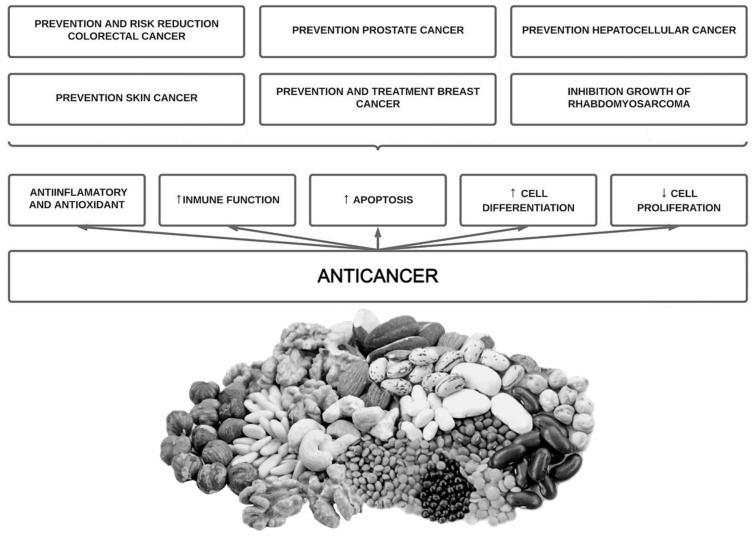
Overview of potential beneficial bioactivities of phytate on cancer.

Phytate has been shown to inhibit the growth of different cell lines in a dose and time dependent manner [117]. This has been seen in hematopoietic cell lines (normal and leukemic) [123,124], in human colon cancer cell lines [119,122], in breast cancer cell lines (estrogen-receptor positive and negative) [125], cervical cancer cell lines [119], in prostate cancer cells [126,127,128], in hepatocarcinoma [129], fibrosarcoma [130] and rabdomyosarcoma [131]. Recently, Markiewicz et al., 2021 [131] investigated the combined effect of phytate and butyrate (PA1B1) on cell lines derived from cancer (HCT116 and HT-29) and healthy (NCM460D) human colonic epithelium. They found that phytate and butyrate together enhanced their pro-apoptotic effect in cancer cells, whereas in the healthy cells phytate suppressed the pro-proliferative action of butyrate and activated a pro-survival pathway [132]. Phytate could exhibit a specificity for cancer cells, being a drug with a selective action [72]. An overview of phytate intake potential benefits on cancer is shown below (Figure 3).

### 7.2. Animal Studies

Phytate effectivity to reduce neoplastic activity has been tested/demonstrated in several animal models. One of the most researched areas is that of colon cancer. Different carcinogens were given to rats and mice, and phytate was administered by food or mixed with water with promising results. Phytate reduces the mitotic rate in the colonic crypts of the animals [133,134,135], and increases chelation of dietary iron, reducing the promotional phase of carcinogenesis [136], and reducing the formation of abnormal crypts [137,138,139], increasing cell apoptosis and differentiation [140,141] and favorably affecting colon morphology [140,141].

These results were not limited to the colon tissue, as other studies using different experimental models have showed the effectiveness of InsP6 in other tissues. In liver tissue, InsP6 could inhibit tumorigenicity and suppress/regress the growth of HepG2 cells in a transplanted mouse model [142]. Similar results were obtained using a hepatocellular model [143]. In the lungs, phytate showed the reduction in pulmonary carcinogenesis [144,145]. In mammary tissue, some studies reported a 19% reduction in the incidence of mammary carcinoma when InsP6 and inositol were used together [146]. Tumor number, multiplicity and tumor size were also reduced by the use of InsP6 plus inositol [147]. In a skin carcinogenesis model, the animals consuming InsP6 during the initiation stage showed an approximately 50% reduction in the mean number of papilloma per animal [148]. Phytate intake inhibits growth of rhabdomyosarcoma in a dose and time dependent manner. When the study was extended to five weeks, a 49-fold reduction in tumor size was observed in mice treated with InsP6 [131]. For the first time, in 2006, in a trial using in vivo mouse and in vitro human prostate cells, phytate was seen to repress telomerase activity in mouse and human prostate cancer in a dose-dependent manner [149]. It is worth mentioning that, in different studies, phytate effectiveness is reported as dependent on dose, given either before or after the carcinogen administration [117].

Phytate is abundant in cereals and legumes. Vucenick et al., 1997 [150], carried out a comparative trial on rats to investigate whether the positive anti-cancer effects reported with high phytate intake were due to the bran intake or the phytate intake. They established five groups: one without phytate (AIN-76 diet); AIN-76A diet containing 5%, 10%, or 20% Kelloggs’ All Bran; the fifth group received 0.4% InsP6 in drinking water (an equivalent to the InsP6 content in 20% bran). Tumor incidence was reduced by 16.7%, 14.6%, and 11.4%, respectively. Only the rats given 0.4% InsP6 in drinking water, equivalent to that in 20% bran, had a 33.5% reduction in tumor incidence, reaching statistical significance. Results showed that the effects were inherent to InsP6 intake [150].

### 7.3. Epidemiological Studies in Humans

Lifestyle interventions are essential in reducing cancer incidence. Although dietary factors are thought to be important in determining the risk of developing cancer, establishing the exact effects of diet on cancer risk has proved challenging [151,152,153,154]. In an umbrella review recently published by Papadimitiu et al., 2021 [155], calcium, dairy, and whole grain products were associated with a lower risk of colorectal cancer [155]. Additionally, the intake of fruits and vegetables were inversely associated with head and neck cancer risk [155]. Some research has showed that the Mediterranean diet, DASH diet and other similar diets have a protective effect against cancer development [151].

The intake of Mediterranean foods (such as fish, vegetables, whole-grains, legumes, nuts, seeds and fruits) contributes to a reduction in the risk of developing cancer, through a series of mechanisms that reduce tumor cell growth, whereas anti-oxidative and anti-inflammatory effects increase chemoprotective effects and inhibit tumor development [151].

In breast cancer, high adherence to a Mediterranean diet decreases incidence by between 6–20% [156], especially in post-menopausal women [157]. In colorectal cancer, high adherence to Mediterranean diet reduces the risk of colorectal cancer by 30% in men and 45% in women [158]. In prostate cancer, a high adherence to Mediterranean diet was not only inversely associated with a low incidence of prostate cancer, but it was also associated with lower cancer malignancy and mortality rate in patients without metastasis [159,160]. Similar results have been replicated in other malignancies such as gastric cancer [161], bladder cancer [162], head and neck cancer [163], pancreatic cancer [164] and in lung cancer, especially in heavy smokers [165]. It is worth mentioning that being overweight or obese increases estrogen production and hormonal imbalances, heightening the risk of hormonal-related malignancies such as endometrial cancer [166]. The Mediterranean diet weight loss effect could have a role in reducing hormonal-imbalance-related malignancies.

It is already known that the food matrix may exert a larger effect that any single component of the food by itself [167]. Already in 1985, some authors such as Graf et al. [168] mentioned that high-fibre diets are not always correlated with low frequency of colonic cancer, suggesting the involvement of additional dietary constituents [168]. The authors hypothesized that the antioxidant and anti-inflammatory effects of phytic acid would be responsible for the lower incidence of colorectal cancer, independently of fibre intake.

Some of the foods typically found in the Mediterranean diet have different components in their nutritional matrix that can exert a positive health effect (for example nut, seeds and wholegrains contain omega 3, fibre, phytate, phytochemicals and polyphenols) thus the methodological design of specific studies in the future in order to establish which component exerts which particular effect is an absolute priority.

### 7.4. Clinical Trials in Humans

Cancer treatment has evolved achieving better curation rates and increasing quality of life. However, the overall cure rate remains unsatisfactory. Moreover, the issues caused by the toxicity of oncological drugs remains an issue for the quality of life of cancer patients. New therapeutic options have been introduced to reduce the side effects of cancer treatment. Some nutraceuticals, such as phytate, have been studied as a preventive tool against cancer development, as curative or even as an adjuvant to improve quality of life [169].

In lung cancer, myoinositol but not phytate specifically, in doses ranging from 12 to 30 g/d showed a significant increase in the regression rate of pre-existing dysplastic lesions (phase I [170] and phase IIb [171] studies). The best effects were seen at 18 g, with greater doses showing no further benefits and greater side effects.

Breast cancer is the most common malignancy in the female population globally. Both phytate and the combination of phytate and myoinositol have been studied for their potential benefits in the treatment of breast cancer [116,172]. Patients with ductal invasive breast cancer treated with a combination of phytate and inositol in during chemotherapy showed a significantly higher quality of life with higher functional scores compared to placebo groups [172]. Additionally, no leucocyte or platelet drop was observed in the group treated with this nutraceutical combination, outcomes which would be important in order to minimize hematological complications and susceptibility to infections [172]. Proietti et al., 2017 [116], in a double-blind randomized control trial conducted over 6 months, used 200 mg of topical InsP6 in patients with ductal breast cancer stage II-III postoperative (lumpectomy) daily during chemotherapy [116]. This resulted in fewer side effects, fewer postponed chemotherapy cycles and an improvement in the quality of life and functional status in the treatment group. As seen in previous works, white blood cell count remained at normal values in the InsP6 patients, whereas it decreased drastically in the control group [116].

Colon cancer is one of the most prevalent cancers in the world. Promising results have been observed in vitro and in animal studies using phytate for colon cancer, firstly, it works as a chemoprotective agent preventing side effects [169] and secondly, it shows an immunostimulant effect on NK-cells, which is linked to a reduction in tumor incidence [173,174]. However, these results have not been confirmed in humans.

A single case report [175] showed benefits of the combination of phytate and inositol on melanoma. A patient diagnosed with stage IV melanoma declined traditional therapy and instead tried InsP6 and inositol. The patient achieved complete remission and remains in remission 3 years later. These results have not been replicated.

## 8. Other Phytate Applications

The increased prevalence of antibiotic resistance is a public health issue that makes finding natural ingredients capable of reducing/inhibiting microbiological activity crucial. Phytate has shown its antibacterial properties in vitro on Enterococcus faecalis [176], Escherichia coli O157:H7 [177] and in Bacillus Subtilis [178]. Although the mechanism it is not fully known, the weak acid theory could explain the disruptive effects of phytic acid on the cytoplasm and pH homeostasis [137].

Phytic acid has shown in vitro antiviral effect. Phytate inhibited the cytopathic effect of human immunodeficiency virus, leading to the hypothesis that it may act on early replicative stages [179].

Biscuits enriched with InsP6 [4], capsules containing phytin in order to prevent lithiasis and a mouth wash for plaque prevention [4] are all available on the market.

Studies in basic and translational research have revealed the role of AGEs in the development and progression of various age-related pathological conditions [180]. Sanchis et al., 2018 [31], showed that a three month InsP6 diet significantly lowered the levels of circulating AGEs (~25%) in patients with T2DM. This effect is likely to be explained by InsP6-mediated chelation of Fe3+ [31].

Phytate may have application in the food security industry. Some research has shown that it could be recommended as a food additive which prolongs the stability of both raw and cooked meat, to a larger degree when it is cooked [181]. Moreover, adding InsP6 to wine and other beverages would reduce the side effects and toxicity of high metal content in beverages [182]. The pharmaceutical industry tested the addition of InsP6 to drugs on rats and showed that it was able to improve drug absorption and increase oral bioavailability [183]. In dentistry, InsP6 has attracted attention, due to antimicrobial action producing a caries-preventive effect [184]. It has also demonstrated the ability to bind to hydroxyapatite, forming a monomolecular surface layer that limited both the growth and dissolution of HAP crystals, thus inhibiting caries, plaque formation and enamel dissolution [185]. These findings have led to the development of several patented oral care regimes [185].

## 9. Controversies of Phytate Intake

Bioavailability is a measure of the amount of absorption and supply to cells and organs of nutrients (micronutrients and macronutrients). Any reduction in the bioavailability of nutrients can affect our health. Our bodies do not have endophytase; therefore, phytate cannot be broken down by the human body [186]. Thus, the minerals chelated in phytate are not bioavailable. This finding has led to phytate being tagged as an “anti-nutrient”. Nevertheless, this effect can be reversed by phytase-induced degradation of phytic acid during food processing (by means of phytases present in the plant/flour) as well as during digestion (by phytase activity expressed in microbiota residing in the intestinal tract) [187]. Other techniques are also effective in reducing phytate content [188] as cooking at 95° for one hour reduce phytate content between 11–80% [188], soaking reduce phytate content between 17–80% and sprouting reduce phytate content more than 60% [188].

Several studies showed that phytate intake could reduce mineral bioavailability, especially iron absorption [23,189]. As mentioned by Brouns, 2022, the results in vivo can differ from those obtained in vitro. A decrease in soluble minerals in vitro does not necessarily translate into an increase in bioavailability in vivo because other environmental/dietary factors may play a role [187].

The bioavailability of minerals can be calculated according to the phytate:mineral ratio [188]. The optimal ratio of phyate/mineral could be lower than 0.4:1 (phyate:iron) [190], higher than 15:1 (phyate:zinc) [191] and higher than 0.17:1 (phyate:calcium) [192]. The interaction between phytate and other nutrients must be mentioned. Miller et al. reported that the effect of dietary phytate on zinc absorption when controlling for dietary zinc was very small and not statistically discernable [193]. Additionally, Hope et al., 2019 [194], in a 12-week, randomized, parallel study in humans, compared two different wholegrain bread formulations. One of them contained high phytate content and the other one, low phytate content. The difference in phytate content did not impact iron status. Other researchers observed similar results on iron metabolism when comparing the effect of genetically modified maize containing 30–50% less phytic acid versus regular maize [195,196]. Several human trials indicated too that phytate intake of 2 g per day did not affect mineral balance [31,197,198,199].

The dose and route of administration of phytate are highly variable in the different available in in vivo (animal and humans) studies. The administration of phytate and their benefits are associated with the appearance of InsP6 and its phosphorilated inositols (InsP5, InsP4, InsP3 and InsP2) in the urine [8]. Otherwise, in the absence of oral or topical phytate, only InsP2 is quantifiable in the urine [8]. The best available option to measure InsP6 and their dephosphorylation products in urine are coupling high liquid performance chromoatography (HLPC) with mass spectrometry (MS) [8]. In rats, the administration of topical or oral phytate produce an elevation of phytate and their dephosphorylated products in urine [200,201]. These elevation lasts for 22 days after the cessation of phytate supplementation [200,202]. When healthy volunteers received a high phytate they experienced an increase in urinary excretion of total InsPs that return to baseline levels in 16 days after the cessation of high phytate intake [202]. Thus, the topical or the oral supplementation of InsP6 produced an increased urinary excretion of total InsPs in animals and in humans [8]. However, as Grases et al., 2019 [8], mentioned, many aspects of the chemical analysis of InsPs require further work and it would be very interesting to develop specific analytical method for quantification phytate and the products which result from its dephosphorylation.

Despite the idea that InsP6, due to its high charge density, could not cross the lipid bilayer of cellular membranes [203], Grases et al., 2005, showed the penetrance of phytate in multiple tissues (kidney, brain, bone, plasma and urine) [204]. In fact, the majority of InsP6 present in the organism is of dietary origin and its endogenous synthesis is not important [204]. Moreover, when dietary phytate intake is increased, an elevation in the urine of phytate and its dephosphorylated products is shown, in rats and in humans [8,197,200,201,202]. In fact, the entry of phytate and its derivates through the intestine is believed to be paracellular, as in the case of bisphosphonates, and for this reason the rate of absorption is low. Ferry et al., 2002 [119], in a study examining HeLa cells culture, suggested that InsP6 could enter the cells pinocytotically and then being further dephosphorylated into lower InsPs derivates. Thus, the evidence shows the absorption of InP6 exists in different tissues, although the specific mechanism which InsP6 enters the cell require further work.

The effectivity of phytate intake seen in in vivo, animal and epidemiological data has revealed the need for well-designed human clinical trials to assess the effectivity of phytate intake in vascular calcifications, urolithiasis, osteoporosis, cognitive function, metabolic health and cancer. In Table 1, we show all the clinical trials performed in humans using phytate.

## 10. Conclusions

**Figure 4:**
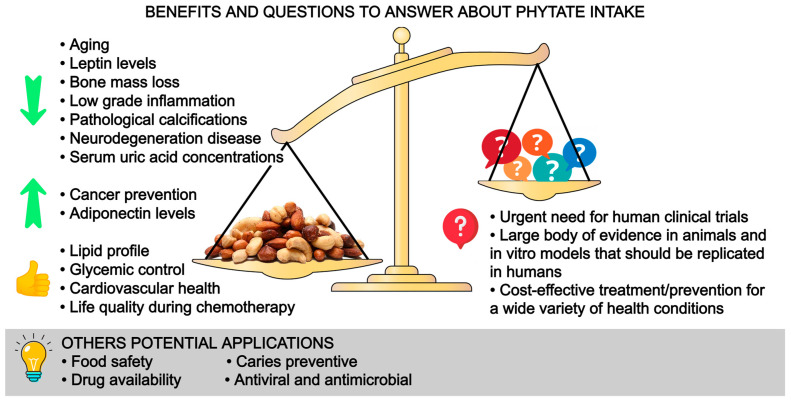
Benefits and questions to answer about phytate intake.

In this review, we have summarized the benefits of phytate on health and we identified questions to answer in the future (Figure 4). In a structured way, we have shown the effects of phytate on vascular calcifications, urolithiasis, osteoporosis, cognitive function, metabolic health, cancer and some potential applications. These are our take-home messages:

- It is well known that phytate is a powerful agent for preventing calcifications in biological fluids: usefulness in renal lithiasis treatment, sialolithiasis and vascular calcifications.
- Phytate can also avoid or disturb loss of bone mass. Low doses of phytate could generate a strongly protective effect as we have seen in osteoporosis where a minimum of 307 mg/day of phytate (1–2 servings of nuts or legumes per day) reduce the risk of osteoporosis.
- Phytate has shown benefits in reducing leptin levels, increasing adiponectin, improving carbohydrate and lipid metabolism, decreasing AGEs (the potential reduction in microvascular and macrovascular diabetes-related complications and aging), improving anti-inflammatory and antioxidant effects and improving quality of life during chemotherapy.
- There is no pharmacological intervention available that directly reduces neurological decline, vascular calcifications, and urolithiasis. Phytate has shown effectivity in these regards in in vitro, animal, and epidemiological data.
- Recommending a diet high in phytate such as the Mediterranean diet or DASH diet can exert multiple health benefits with no harm.
- The effectivity of phytate intake seen in in vivo, animal and epidemiological data has revealed the need for well-designed human clinical trials to assess the effectivity of phytate intake in vascular calcifications, urolithiasis, osteoporosis, cognitive function, metabolic health and cancer. Phytate intake or phytate-based drugs/supplements should be investigated further.

## Figures and Tables

**Table 1:** Clinical trials in humans using phytate.

Year of Publication and Authors	Type of Clinical Trial	Studied Population	Phytate Formulation and Dose Administrated	Duration of the Intervention	Route of Phytate Administration	Outcomes	References
Perelló et al., 2018	Double-blind, randomized, placebo-controlled Phase I study	Healthy and hemodialysis patients	In healthy volunteers the dose was 0.5 mg/kg and ascended to 12.5 mg/kg. In hemodialysis patients was 9 mg/kg	28 days	Parenteral	Safety and tolerability, and lack of significant dialysability of IV SNF472.	[27]
Raggi et al., 2020	Double-blind, placebo-controlled Phase 2b trial	Hemodialysis patients	Patients were randomized 1:1:1 to SNF472 300 mg (n = 92), SNF472 600 mg (n = 91), or placebo (n = 91) by infusion in the hemodialysis lines thrice weekly during hemodialysis sessions	52 weeks	Parenteral	SNF472 significantly reduced the progression of coronary artery calcium and aortic valve calcification in patients receiving hemodialysis	[29]
Sanchis et al., 2018	Single-center, randomized, crossover, open-label study.	Patients living with type 2 diabetes	Diet plan with InsP6 supplementation (1 capsule of 380 mg of calcium–magnesium InsP6 thrice daily) or the same diet plan without IP6 supplementation.	48 weeks	Oral	InsP6 supplements for 3 months significantly reduced levels of circulating AGEs and HbA1c	[31]
Estruch et al., 2018	Multicenter trial randomly assigned (in a 1:1:1 ratio)	High cardiovascular risk patients without cardiovascular disease	A Mediterranean diet supplemented with extra-virgin olive oil, a Mediterranean diet supplemented with mixed nuts, or a control diet (advice to reduce dietary fat.	Median follow-up of 4.8 years	Oral	Mediterranean diet supplemented with nuts (high phytate content) reduces the incidence of major cardiovascular events was lower compared to a reduced-fat diet.	[33]
Conte et al., 1999	Single center randomized trial.	Active calcium oxalate stone-formers.	Potassium citrate, phytate-rich dietary complement or placebo.	15 days	Oral	Reduction in the lithogen urinary risk	[48]
Guimerà et al., 2022	Single center randomized trial.	Patients with hypercalciuria (> 250 mg/24 h) and osteopenia or osteoporosis in the femur and/or spine (determined by densitometry)	1 capsule of 380 mg of calcium–magnesium InsP6 versus placebo.	12 weeks.	Oral	Phytate group had significantly lower calciuria and ß-Crosslaps levels.	[49]
Grases et al., 2009	Randomized, double-blind, three-period crossover clinical	Healthy dental plaque-forming volunteers	Control period (no mouthwash treat- ment), a placebo period (mouthwash with 0.001% zinc) and a phytate-treatment period (mouthwash with 0.001% zinc and 0.1% phytate).	3 weeks each period for 9 weeks total.	Topical mouthwash	High efficacy exhibited by phytate in reducing dental calculus formation.	[50]
Ikenaga et al., 2019	Randomized, double-blind placebo-controlled crossover study.	Asymptomatic hyperuricemic subjects	Placebo or InsP6 drinks (600 mg twice daily).	2 weeks	Oral	Significantly reduced uric acid levels in comparation with placebo.	[100]
Estruch et al., 2006	Substudy of a multicenter, randomized, primary prevention trial of cardiovascular disease.	Asymptomatic at high cardiovascular risk.	A Mediterranean diet supplemented with extra-virgin olive oil, a Mediterranean diet supplemented with mixed nuts, or a control diet (advice to reduce dietary fat.	3 months	Oral	Improved fasting blood glucose, reduced blood pressure and increased HDL/cholesterol ratios in the high phytate diet in comparison with a low-fat diet.	[110]
Toobert et al., 2003	Randomized clinical trial	Postmenopausal women living with type 2 diabetes	Mediterranean Lifestyle Program (Mediterranean low-saturated fat diet, stress management training, exercise, group support, and smoking cessation) versus with usual care.	6 months duration	Oral	Reduction in HbA1c by the high phytate diet.	[111]
Elhayany et al., 2010	Prospective randomized clinical trial	Patients living with overweight and type 2 diabetes	A low carbohydrate Mediterranean, a traditional Mediterranean and the 2003 American Diabetic Association diet were compared.	12-month period	Oral	Both high phytate diets showed better weight loss effects and better reduction in HbA1c compared to other diet	[112]
Mirmiran et al., 2019	Randomized crossover clinical trial	Patients living with type 2 diabetes	Compared a legume-free diet or a legume-based diet.	8 weeks	Oral	Legumes (which are high in phytate) increased serum adiponectin concentrations	[113]
Lam et al., 2006	A phase I, open-label, multiple dose, dose-escalation clinical study	Smokers	A dose escalation study ranging from 12 to 30 g/d of myo-inositol for a month	1 month	Oral	Myo-Inositol in a daily dose of 18 g for 3 months is safe and well tolerated.	[178]
Lam et al., 2016	A randomized, double blind, placebo-controlled phase IIb study	Smokers with ≥1 site of dysplasia identified by autofluorescence bronchoscopy-directed biopsy	Placebo or myo-inositol 9 g once a day for 2 weeks, and then twice a day.	6 months	Oral	Reduction in progressive disease rates and IL-6 levels.	[169]
Bacic et al., 2010	Prospective, randomized, pilot clinical study.	Patients with invasive ductal breast cancer who received polychemotherapy.	InsP6 + Inositol (6 g) in comparation with placebo (vitamin C)	6 months	Oral	Significantly improved patients quality of life and protected patients from the reduction in the number of leukocytes and platelets.	[170]
Proietti et al., 2017	Double-blind and randomized controlled trial	Patients with breast cancer 6 weeks after lumpectomy.	4% topical skin formulation of InsP6 once a day versus placebo (gel containing hyaluronic acid).	6 months	Topical skin	Topical InsP6 significantly improved quality of life and functional status reducing side effects compared to control group. Moreover, white blood cells and platelets count values where higher in the treated group.	[171]
Sanchis et al., 2022	Randomized and crossover trial	Patients living with T2DM	1 capsule of 380 mg of calcium–magnesium InsP6 thrice daily	12 weeks	Oral	Significant decrease serum levels of HbA1c and increase adiponectin levels.	[114]
